# Knowledge about HPV vaccination among Croatian university students: Ivan Miskulin

**DOI:** 10.1093/eurpub/ckac131.394

**Published:** 2022-10-25

**Authors:** I Miskulin, I Simic, Z Pavic, M Miskulin, V Bilic-Kirin, J Kovacevic

**Affiliations:** 1 Department of Public Health, Faculty of Medicine Osijek, University of Osijek, Osijek, Croatia; 2 Department of Sociology, Faculty of Humanities and Social Sciences, University of Osijek, Osijek, Croatia

## Abstract

**Background:**

Studies have shown the low uptake of the human papillomavirus (HPV) vaccine among university students. Knowledge about the HPV virus and the vaccine can be a factor that may sway an individual’s decision to receive the vaccine. This study aimed to investigate HPV vaccine uptake and knowledge about HPV vaccination among Croatian university students.

**Methods:**

This cross-sectional questionnaire study was conducted from February to May 2021 period. A validated, anonymous questionnaire that contained questions regarding demographic data, data about HPV vaccine uptake, and data regarding knowledge about the HPV virus and the vaccination was self-administered to a cross-faculty representative student sample of the University of Osijek in Eastern Croatia.

**Results:**

The study sample included 840 subjects with, median age of 20 years (interquartile range 20-21), 45.8% males, and 54.2% females. The prevalence of vaccination uptake in the studied population was 20.8%. The study revealed that there were 25.6% of students with a low level of knowledge and 74.4% of students with satisfactory levels of knowledge about the HPV virus and the vaccination. The excellent knowledge about the HPV virus and the vaccination was more frequently shown by students who studied in biomedicine and health area of science (p < 0.001) and students with an excellent average grade of study (p < 0.001). HPV vaccination uptake was higher among females (p < 0.001), students who studied within biomedicine and health area of science (p < 0.001), students with an excellent average grade of study (p < 0.001), and students who showed excellent knowledge about the HPV virus and the vaccination (p < 0.001).

**Conclusions:**

The majority of Croatian university students had a satisfactory level of knowledge about the HPV virus and the vaccination but the vaccination uptake is still very modest. Additional efforts are needed to organize more appropriate education and promotion of vaccine uptake in the studied population.

**Key messages:**

• The HPV vaccination uptake among Croatian university students is very modest although the majority of students have a satisfactory level of knowledge about the HPV virus and the vaccination.

• Continuous examination of HPV knowledge gaps and identification of factors influencing vaccine uptake is key to increasing vaccination rates in the Croatian university student population.

